# A literature review on operational decisions applied to collaborative supply chains

**DOI:** 10.1371/journal.pone.0230152

**Published:** 2020-03-13

**Authors:** Enrique Holgado de Frutos, Juan R. Trapero, Francisco Ramos

**Affiliations:** 1 Department of Business Administration, University of Castilla-La Mancha, Ciudad Real, Spain; 2 Department of Electrical, Electronics, Control, and Communications Engineering, University of Castilla-La Mancha, Ciudad Real, Spain; Shandong University of Science and Technology, CHINA

## Abstract

Throughout the last decades, collaborative schemes, under an amalgam of different acronyms (ECR, CPFR, VMR, etc.), have been developed to mitigate the problematic Bullwhip effect. Essentially, companies work together by either sharing information, making joint decisions, or sharing benefits to reach potential synergies. This work aims at reviewing these works through a systematic literature review process to investigate the different collaborative models from an operational perspective. A total of 92 articles have been classified into 3 categories: Information Exchange; Vendor Managed Replenishment; and Synchronized Supply Chain. For each category, we have identified the type of research, supply chain structures, forecasting models, demand characteristics, replenishment policies and assumptions employed in the considered articles. This article identifies the main results achieved and the gaps and opportunities to be developed as further research.

## 1 Introduction

Global competition has forced companies to be everyday best-in-class in all fields they work on. Nowadays, it does not matter whether a company develops and owns the patent of the ultimate product: if it is not part of a well-designed and efficient supply chain, it can end up drowning in costs [1] and, surely, struggling to be profitable since competitive firms will develop a substitute counterpart [2]. According to the American Production and Inventory Control Society (APICS) dictionary [3], a supply chain can be defined as *“The global network used to deliver products and services from raw materials to end customers through an engineered flow of information, physical distribution, and cash”*. Therefore, Supply Chain Management (SCM) is of paramount importance and companies have been investing large amounts of resources to enhance it in the last decades.

SCM has been broadly studied from the first time it was mentioned in the literature to manage supplies for the US Army during the II World War [4], to recently, when SCM companies analyze the new trends and challenges as Internet of Things (IoT) [5], Big Data [6] or Circular Economy [7]. SCM built upon the Logistics framework [8], seeks to achieve connections and collaboration [9] between the processes of the entities that are part of the same supply chain looking for a common benefit.

Among the SCM challenges, one of the most fascinating problems is the Bullwhip effect, which can be defined as the demand variability amplification as one move upwards in the supply chain. These fluctuations reduce supply chain effectiveness, decrease the service level and raise costs [10–12]. The first time Forrester, the forefather of the debate in the literature for over six decades, mentioned this phenomenon dates back to 1958 [13, 14], but the term Bullwhip effect (BWE) or Forrester effect was not coined until 1997 by [16]. Note that Forrester effect [14] is more related with the Demand Signal Processing called in the past Demand Amplification. For the interested reader, see reference [15]. Sometimes, it is also referred as “the first law of supply chain dynamics” [11]. This effect and its consequences have been subject of numerous studies [16–21].

BWE is very related to the uncertainty that takes place in a company when executes its own processes, keeping in mind only the information they have in their stand-alone Enterprise Resource Planning (ERP) system within the supply chain [22]. It is considered one of the major causes of inefficiency in SCM [23]. BWE has pernicious effects such as: excessive inventory investments along the supply chain, lower customer service, lost revenues due to shortages, and reduced productivity of capital investment, amongst other inefficiencies [24]; and causes losses of up to 30% of manufacturing profits [17]. Surveys of the BWE literature can be found in [22, 23]. Giard and Sali [23] study 53 articles between 1997 and 2011 and explore 13 axes of analysis such as supply chain structure or demand and inventory control models in order to analyze the causes of the BWE in the productive part of the automotive industry supply chain. Wang and Disney [22] cited 150 articles out of 455 papers that were reviewed. In that reference, the methodologies employed in BWE research were categorized into: empirical, experimental, analytical and simulation-based. The influence of demand, type of forecasting technique, time delay and information sharing were explained. Nevertheless, although it is concluded that Supply Chain Collaboration (SCC) is beneficial, none of the previous surveys have analyzed, in detail, different SCC schemes apart from the Information Exchange (IE) type. Disney and Lambrecht [25] report in an 80-page monograph, empirical evidence of the BWE effect in different industries and its causes, focusing on demand signal processing and lead time within the operational causes. They also analyze the costs that are related to the BWE and the methodological alternatives with different levels of coordination between echelons.

This work extends previous surveys by including more complex versions of SCC. According to [26], SCC is possible by sharing knowledge, information, profits, and risk. Collaboration should be a mutual goal that goes beyond a written contract. Essentially, SCC helps reduce the uncertainty generated [12, 16, 27] and the risks associated [28], and contributes to improve performance [29] along the supply chain, as well as, mitigates BWE [30].

Given the importance of SCC, several reviews on the topic have been recently published. De Almeida et al. [31] provided a review about the impact of behavioral aspects such as trust and collaboration in the SCM, and how those aspects help reduce the mitigation of the BWE. Singh et al. [29] advance on understanding the concept of SCC, where driving forces and barriers are identified. Olson and Xie [32] compare different supply chain inventory systems evaluating the use of information technology, the degree of coordination and identifying the decision responsibility. They also focus on Vendor Managed Inventory (VMI) simulation studies by analyzing 13 articles. Nonetheless, they do not look into operational decisions as either the forecasting or inventory control technique employed.

Unlike previous reviews, the aim of this paper is to provide a survey about how the diverse collaborative schemes have been implemented from an operational perspective and its relationship with the BWE. The reason behind the proposed state of the art is to serve as a guide for both researchers/practitioners interested in either reproducing or implementing a SCC. In other words, the present work intends to answer questions such as: which forecasting/inventory control technique is more utilized for each collaboration scheme?, what are the main results of empirical works?, which assumptions employed the different forecasting and inventory control models?, what kind of supply chain structures have been analyzed?, etc. In general terms, this review will be focused on technical aspects that help summarize the efforts that researchers/practitioners have made in the implementation of SCC schemes, as well as, identifying the main gaps and trends found to guide further research.

In order to structure the work, this review is based on a slight modification of previous classifications of SCC types carried out by [33] and [34]. In addition, as previous reviews in [29, 31], this study adopts a systematic literature review methodology [35].

## 2 Classification of collaborative models

SCC is a relatively new research concept in SCM. First articles date back to mid-1990s when Whang [36] described a VMI model. After that, the Voluntary Inter-industry Commerce Standard Association described a nine steps Collaborative Forecasting and Replenishment model, lately called Collaborative Planning Forecasting and Replenishment (CPFR) model [37].

Essentially, the task of classifying the different collaboration initiatives is a complex one, given the variety of acronyms present in the literature. Authors in [33] assure it is not easy to link external sources of information into vendor production and inventory control when there is more than one partner and they provide information with different level of detail [38]. Holweg et al. [34] also consider that SCC is a superficially simple but deeply complex concept and not as well-defined as it could be expected [39, 40].

There are many business dimensions where two companies could collaborate [29], but forecasting and inventory replenishment are key ones to improve the firms’ performance [41]. In this respect, a simple framework of different collaboration supply chain configurations was defined [33], from no collaboration using customer orders as the only external information sources (Type 0—Traditional supply chain) to sharing the distribution requirements upwards (Type IV—Replenishment, forecasting, customer inventory management and distribution planning). Later, a refinement of that classification was elaborated by [34]. Olson and Xie [32] employed 5 types of coordinated supply chain inventory management systems: traditional supply chains, Efficient Consumer Response (ECR), VMI, Continuous Replenishment (CR) and CPFR.

In this article, we employ the classification proposed by [34] with a slight variation in the representation. Basically, Holweg et al. [34] distinguish 4 different types of collaboration depending on the dimension (forecasting or inventory management) they collaborate, such as: i) The traditional supply chain or the decentralized system with no collaboration; ii) IE, where companies collaborate in terms of improving forecast performance; iii) VMR, where the collaboration is focused on replenishment; and finally, iv) SSC, where forecasting and inventory management is enhanced on the basis of mutual collaboration.

**Fig 1:**
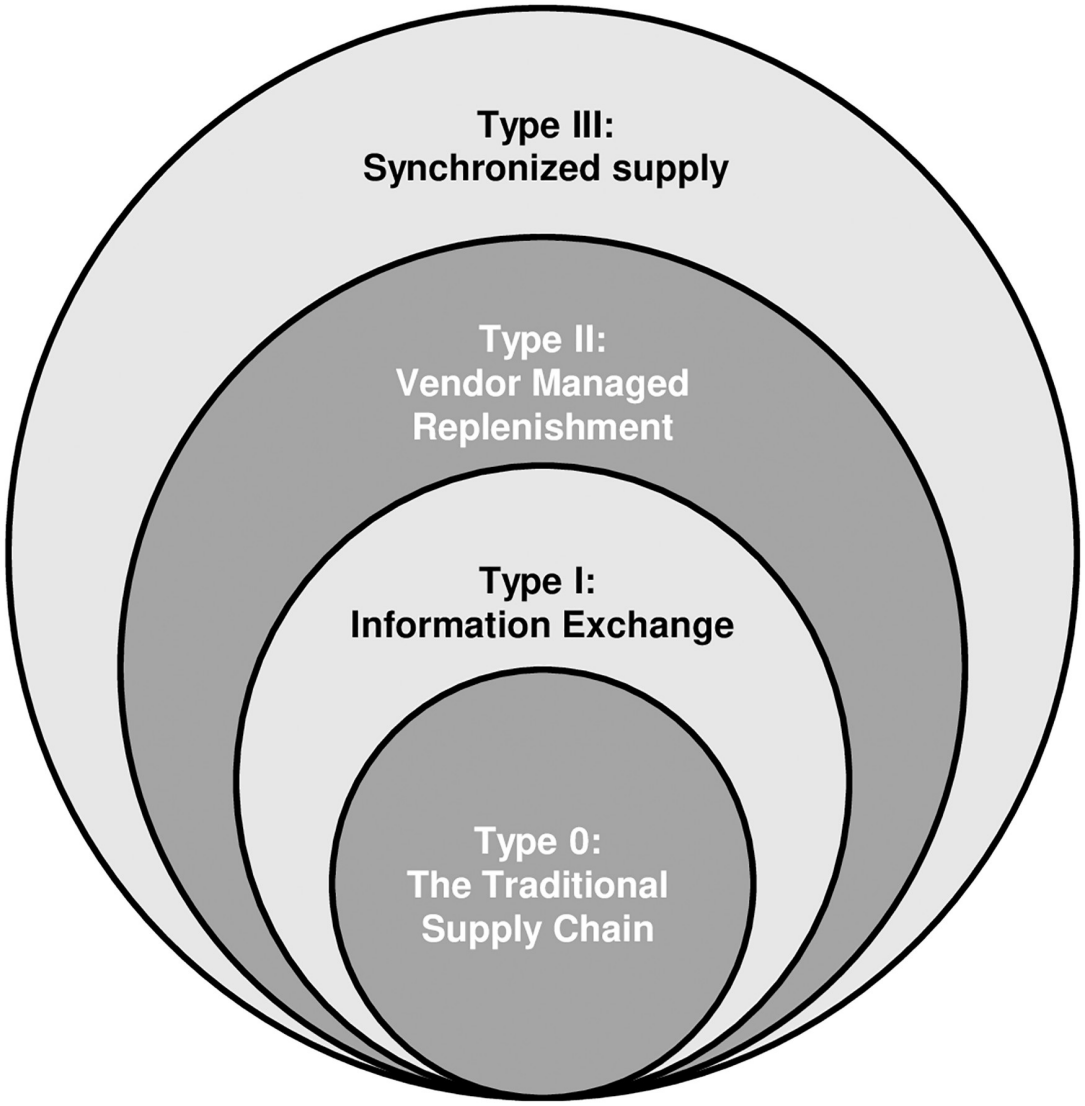
Layered hierarchical collaborative models classification.

In this work, we propose that the different aforementioned types cannot be seen as isolated processes but as the result of different layers of collaboration, and hence, we reformulate the collaborative mechanism types in a layered hierarchical structure as shown in Fig 1. The reason behind this change is to make the classification more flexible to incorporate some articles that they did not fit well in the original form. In particular, according to the classification in [34], IE collaboration improves forecasting performance on the basis of sharing information, however, articles as [42–44], which were published after initial classification was proposed in [34], share information to improve inventory processes instead. Furthermore, since the inventory policy is decentralized cannot be seen as a VMR type. Theferore, with the novel layer visualization of the Holweg and collaborators [34] classification, we relax the assumption that the IE collaboration type is only intended for planning collaboration and it can be also used for decentralized inventory collaboration. This new visualization also remarks that more advanced collaborative mechanisms comprise the less advanced counterparts. In other words, a VMR collaborative mechanism cannot be adopted unless an IE also exists. Therefore, SCC can be divided in four different levels and lower levels are included in upper levels.

The review of the literature will be based on such a classification, where reviewed articles will be included in one of the four types.

## 3 Bibliometric analysis

Several previous reviews such as [31] and [29] followed a systematic literature review process. This survey is also addressed by using a systematic literature review [35]. Note that such a type of review adopts a replicable, scientific and transparent process to overcome potential lack thoroughness of traditional narrative reviews [35]. To carry out the systematic review, a content analysis approach is proposed since it has been employed in many review papers within the supply chain management literature ([45–47] and references therein). The review systematically conducted is based on the following four-step iterative process [45]:

1. **Material collection**, structured process of searching and delimitation of the articles.
2. **Descriptive analysis**, which provides general characteristics of the studied literature.
3. **Category selection**, aiming to construct a classification framework based on a set of structural dimensions and analytic categories.
4. **Material evaluation**, which analyses articles based on the proposed classification framework and interprets the results

### 3.1 Material collection

The bibliography collection ranges from 1997 to 2019 on Scopus Database (www.scopus.com) and only considers articles written in English. Although before 1997, there were articles available that dealt with BWE and collaboration models separately, the first time both concepts were jointly investigated was with the seminal paper of [16]. The final database search was done on March, 5^th^ 2019.

To select the main articles, it is crucial to identify an effective set of keywords that capture the synthesis of the existing literature related to *supply chain collaborative models* and *Bullwhip effect*. These subjects are widely studied but, unfortunately, under an ample variety of creative names and acronyms. Regarding SCC, the keywords searched in the title, abstract or keywords were: *supply chain collaboration, collaborative model, collaborative supply chain, supply chain coordination, information sharing, IE, supply chain integration, VMI, VMR, CPFR, ECR*.

In relation to BWE, we proceeded analogously with the following keywords: *Bullwhip effect, Forrester effect, bullwhip, bullwhip-effect*. See S1 Appendix for the detailed search commands employed. The initial search resulted in a total of 404 papers. A first filter was applied by using an inclusion/exclusion criterion [35]. Such a criterion was based on number of cites: for an article to be considered it would need to be cited at least 20 times in the set of 404 articles. It means that, at least 5% of the articles related with the subject and accomplishing the criterion had considered relevant the article.

This filter was applied by using the VOSviewer tool (version 1.6.10) [48], a software tool that creates connected information maps. A map can be created using items, which are objects of interest. Items may be publications, researchers, or terms. In our work, we have used publications as items. Items can be connected by co-authorship, co-occurrence, citation, bibliographic coupling, or co-citation links. A link is a connection between two items, and each link has a strength that may indicate the number of cited references two publications have in common. Items and links together constitute a network. To construct a network, bibliographic database files (i.e., Web of Science, Scopus, Dimensions, and PubMed files) and reference manager files (i.e., RIS, EndNote, and RefWorks files) can serve as input to VOSviewer. A map also shows clusters that group a set of items. For the interested reader, further information can be found in [48–50].

**Fig 2:**
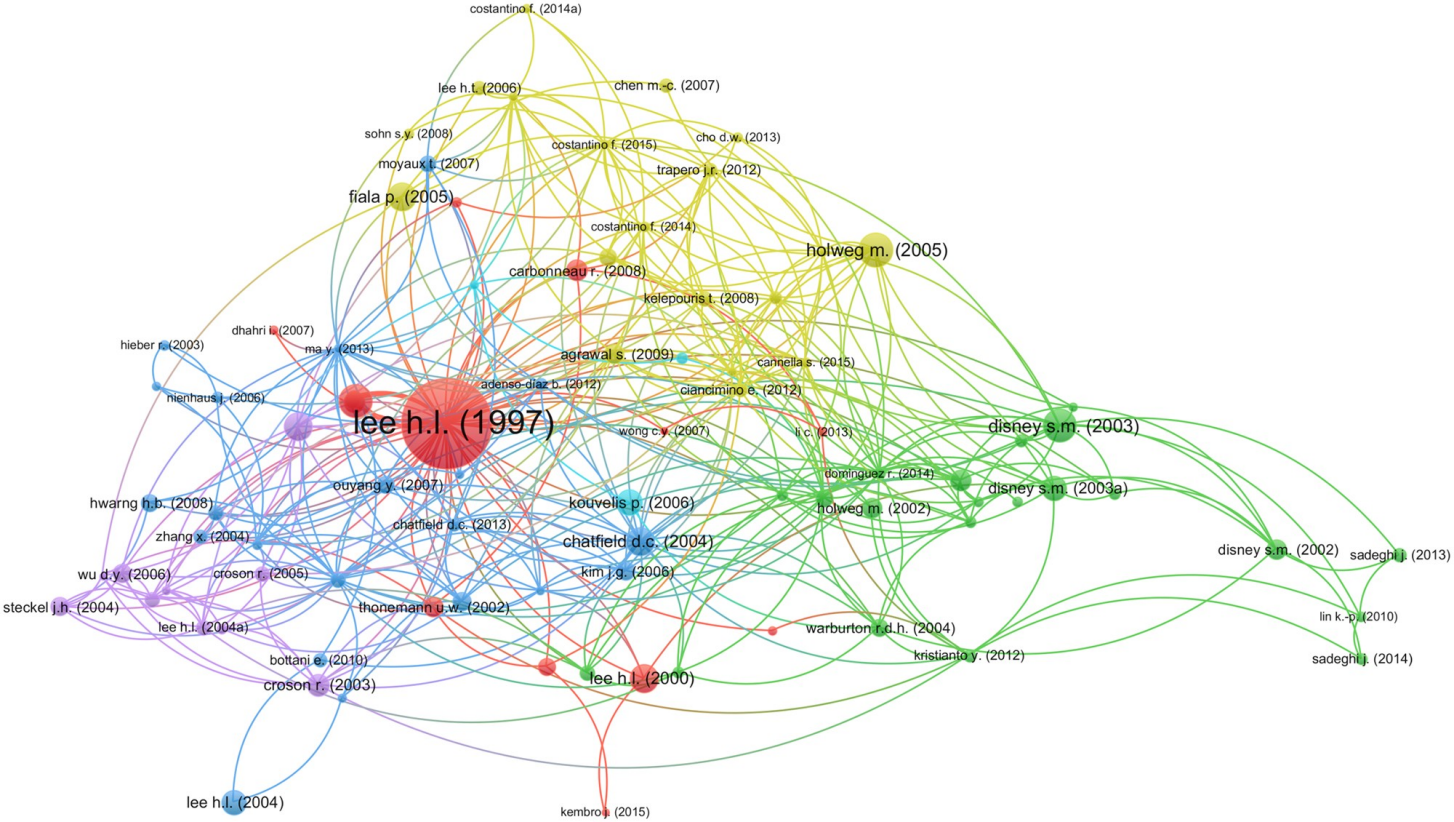
Vosviewer output of the inclusion/exclusion criteria.

VOSviewer provides three different visualizations of the map created, referred to as the network, overlay and density visualization. In this work, we have chosen the network visualization, see Fig 2. The relevance of articles in that figure is proportional to the size of the circle and the label that represents it. Note that the seminal work of [16] stands out from the rest. Furthermore, the extent of relatedness between articles is represented by the distance between them. For instance, see how close are [51] and [52]. Finally, the program automatically organizes the articles according to clusters by using different colors. It brings a total number of 95 articles.

### 3.2 Descriptive analysis

After reading these 95 papers, three of them were excluded because they did not implement any collaborative mechanism [53–55]. Therefore, a total of 92 articles were finally studied and classified. S1 Table shows the selected articles organized by importance according to the number of citations.

**Fig 3:**
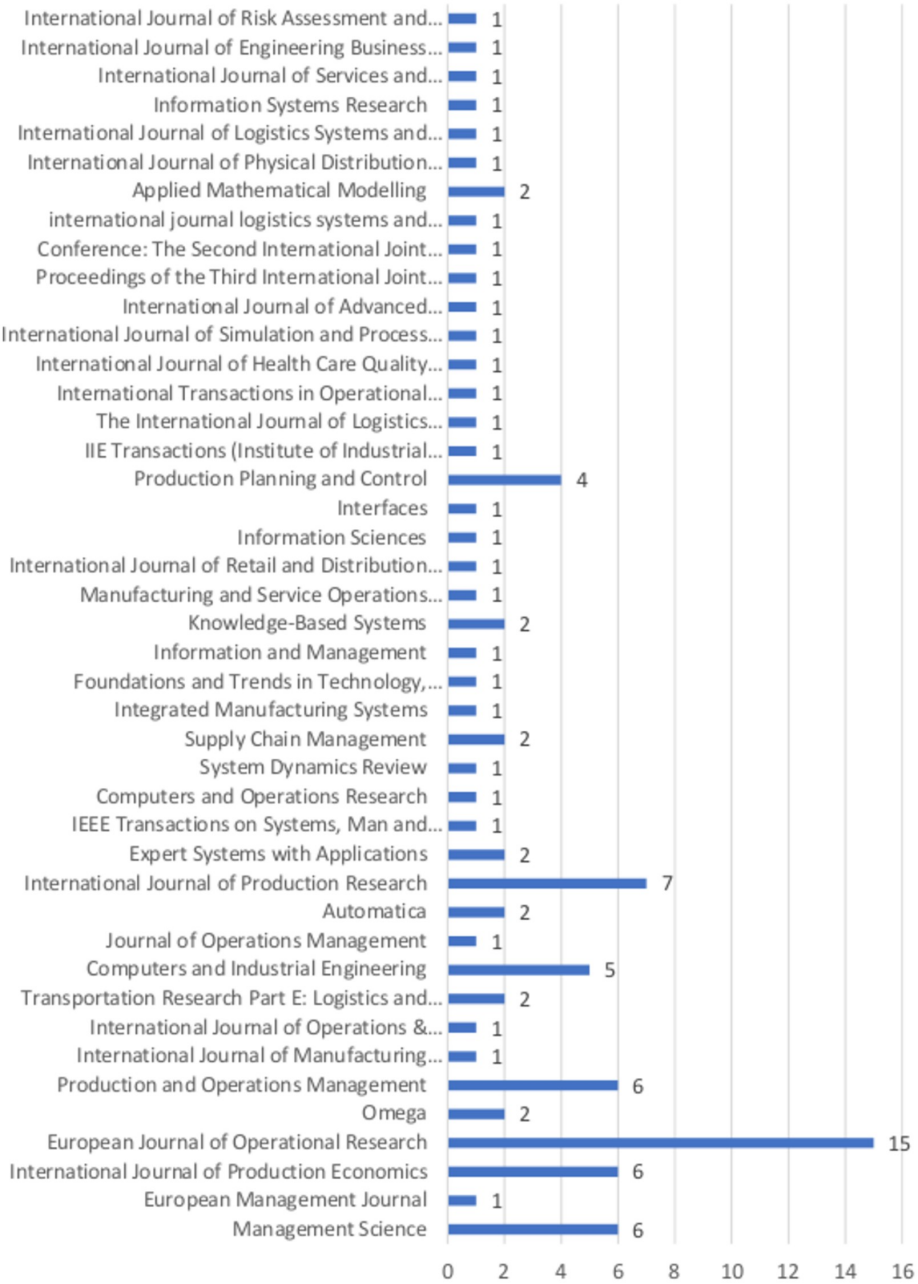
Distribution of reviewed articles per journal.

They spread across 43 different journals, in which 14 published more than one paper. Fig 3 illustrates the distribution of the articles by publication. The high number of international journals where articles have been published and the variety of fields of these journals, indicates that SCC and BWE are actively studied from different perspectives.

### 3.3 Category selection

This step aims to find a framework formed by structural dimensions and analytical categories that allows to organize the literature review. Table 1 shows the 10 structural dimensions that have been accounted for and their possible values. The first dimension refers to intercompany integration. Here, we have used the classification of collaborative types described in Section 2.

**Table 1:** Structure dimensions and analytic categories of the literature review.

Structural Dimension	Analytic categories
Intercompany integration	Traditional Supply Chain
	Information Exchange
	Vendor Managed Replenishment
	Synchronized Supply Chain
Type of research	Empirical
	Theoretical
	Descriptive
	Behavioural
Number of echelons	2, 3, 4+
Supply chain structure	Serial
	Convergent
	Divergent
	Network
Type of demand	Simulated
	Real
Forecasting model/method	ARIMA (AutoRegressive Integrated Moving Average)
	Moving Average
	Exponential Smoothing
	Linear Regression
	Machine Learning
Inventory control technique	Classical OUT
	Control Engineering
	Beer Game
	EOQ
	JIT
Inventory model assumption	Lost sales
	Backordering
Collaboration objective	BWE reduction
	Improve communication and reduce uncertainty
	Improve customer service
	Reduce inventory level
	Reduce costs
Bullwhip effect	Reduced
	Non reduced
	Statement of facts

The type of research describes how the article has addressed the problem. Here, we can distinguish 4 types that are not mutually exclusive: empirical, theoretical, descriptive and behavioral. Empirical works are those whose results are based solely on real data. Theoretical works include both analytical and simulation results. Within theoretical works we have also included articles based on a control engineering perspective. Descriptive articles explain the phenomenon and main characteristics as well as how to solve the described problems from a qualitative point of view. This structural dimension can be seen as a mixed of descriptive and prescriptive articles, [23]. Behavioral category is centered on cognitive limitations of the decision makers, failure to adequately account for feedback effects and time delays, mistrusts and counteracting strategies [56, 57]. Different business games are commonly used under this structural dimension, e.g., the well-known Beer Game.

Number of echelons and supply chain structure dimensions provide an idea of how complex the supply chain under study is. Serial SC consists of echelons only connected to another single echelon upwards and downwards, [23]. In a divergent structure each echelon can be connected to several downstream echelons, although to a unique upstream echelon. Symmetrically, a convergent structure assumes that each echelon can be connected to several upstream echelons and to a unique downstream one. Finally, a network structure considers that an echelon can be connected to any number of upstream and downstream echelons without a cyclic relationship, [23].

Type of demand refers to either the demand has been simulated with random number generators or is based on real data. Note that articles using real demand data that has been fed into simulations are also categorized as theoretical works, in order to distinguish them from fully empirical works.

Forecasting model/method explains how the forecasts are performed. Well-known forecasting models and methods are chosen, but also, Machine Learning approaches, since they are gaining more attention from the SC community.

Inventory control technique dimension classifies the reviewed articles depending on whether the replenishment policy follows a classical Order Up To (OUT) [58]; control engineering approaches; ordering rules typically used in simulated games as the Beer Game; Economic Order Quantity (EOQ); and Just In Time (JIT) ordering types. It is also investigated if the inventory control assume a lost sales case or total backordering.

The two last rows detail the collaboration objectives and whether the collaboration has reduced the BWE, respectively. Although one of the main objectives of the collaboration is to reduce the BWE, such a reduction does not have a direct translation in cost/customer service terms. Therefore, we have complemented the BWE with the following objectives: improve communication and reduce uncertainty (which is associated to an improvement of forecasting accuracy), improve customer service, reduce inventory level, and reduce costs. Note that, statement of facts refers to the case when a straight answer about the reduction of BWE cannot be deduced and other circumstances should also be attended.

To structure the article, next sections analyse the dimensions defined in Table 1 according to the intercompany integration.

As a visual aid to better understand different collaborative models, we retrieve the “water tanks” models utilized by [33, 34] that represent the way two companies collaborate each other in supply chains. Basically, there are two ordering decisions (the “ball-cock valves”) to describe a simple two-level supply chain. Water stored in the tanks represents the inventory level and the water flow indicates product sales.

**Fig 4:**
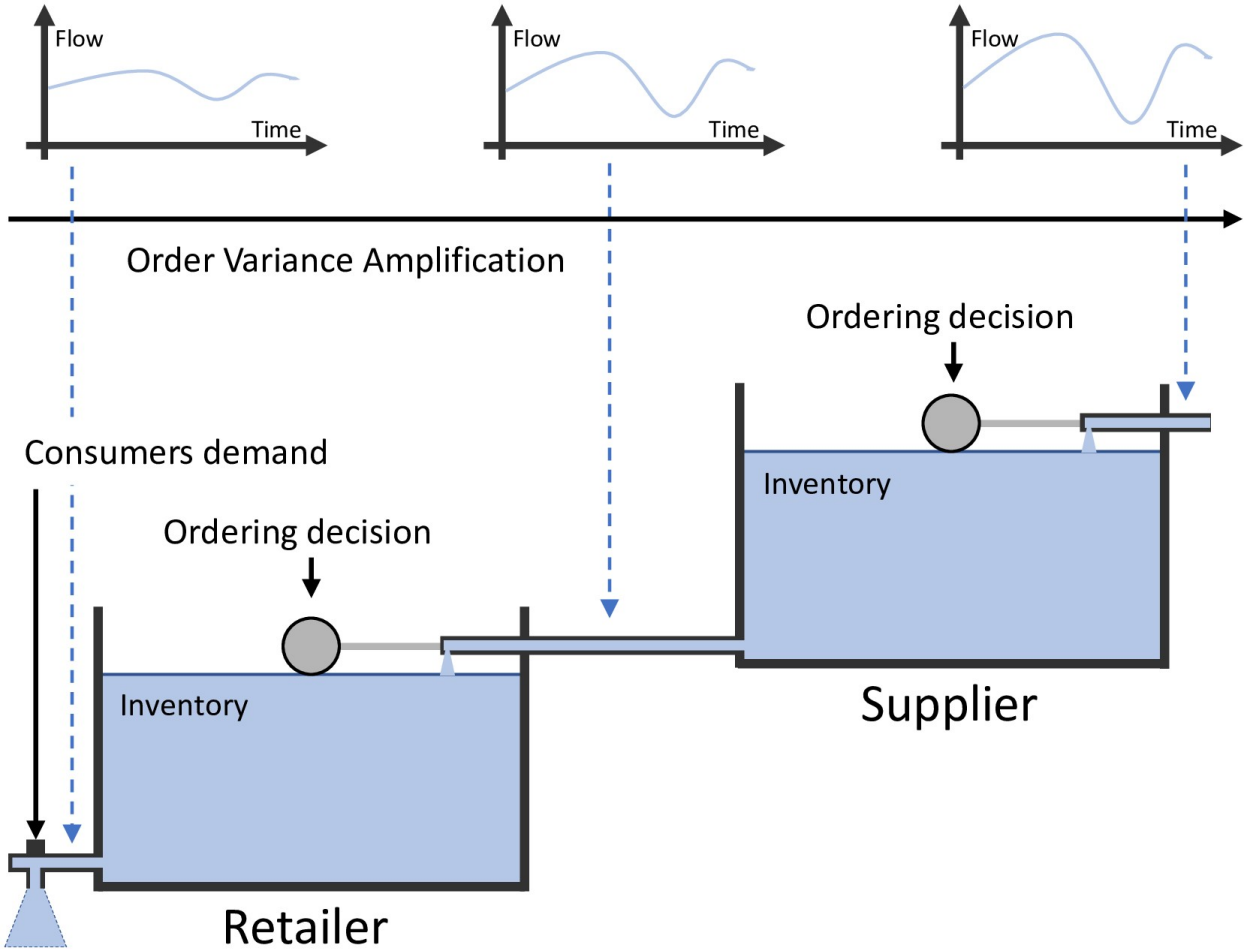
Type 0: The traditional supply chain.

Fig 4 shows the traditional supply chain that refers to a decentralized system where each member feeds its own Forecasting Support System with incoming orders from direct customers [59]. No company considers the situation either up- or downstream of the supply chain. Nowadays, this configuration is still very common. Thus, no collaboration exists between the retailer and the supplier [34] and the BWE problem is expected upwards in the supply chain. Upper part of Fig 4 shows how the flow variance, which represents the order variance, is amplified when moving from retailer to supplier.

This configuration is usually employed as a benchmark when showing the advantages/disadvantages of any other type of SCC, see for example [43, 60, 61].

## 4 Type I—Information exchange

**Fig 5:**
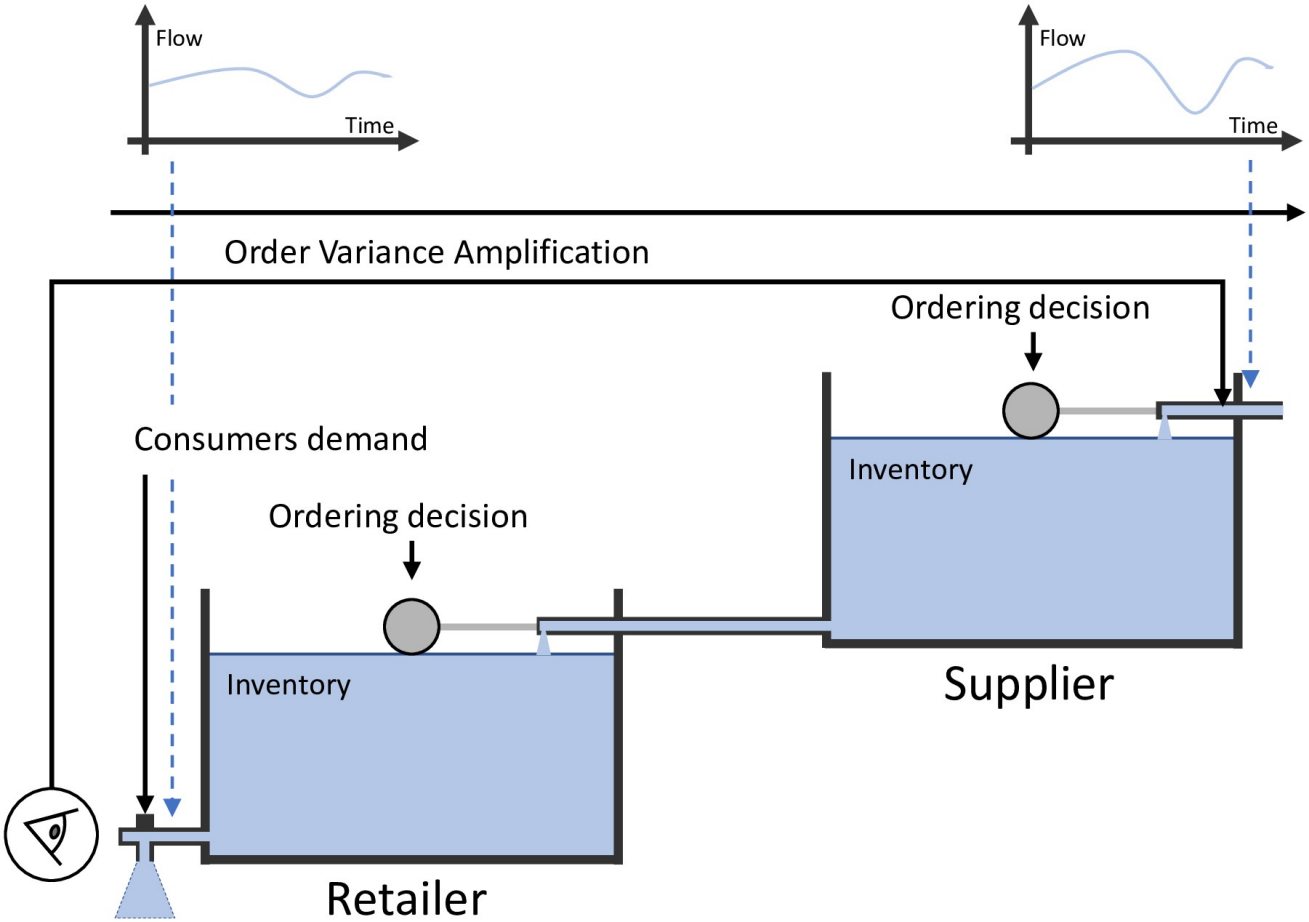
Type I—Information exchange.

Also called information sharing, this collaboration type refers to the extent to which a firm shares a variety of relevant, accurate, complete, and confidential information in a timely manner with its supply chain partners [62], whilst both retailer and supplier order independently looking for improving their forecast for capacity and long-term planning. Fig 5 depicts the water tank analogy for IE, where the supplier is able to “see” the consumers demand in real time, although the ordering decisions are decentralized. The upper plots of flow vs time show that still exists a demand variance amplification, since IE cannot remove completely the BWE.

It is considered the basis of the integration [63], i.e., the essential one required for all further collaboration [62, 64].

Information sharing is the first level of collaboration between companies, see Fig 1, S2 Table summarizes the articles included under the IE scheme. Note that IE is the best documented type of collaboration with 73 out of 92 articles, that is approximately 79% of our sample.

Nowadays, the continuous advancements on Information and Communication Technologies (ICTs)—ranging from the well-known Electronic Data Interchange (EDI) [65] to Big Data [66] and IoT [67] trends—facilitate the information sharing collaboration type from a technological perspective. However, the question of how the internal analytics should be changed, does not have a straightforward answer.

### 4.1 Type of information exchanged

Regarding the information exchanged, Lee and Whang [63] describe the different types of shared information as inventory level, sales data, order status, sales forecast or others. When analyzing the articles that implement an information sharing collaborative scheme, the most frequent piece of information shared is the customer sales data [59, 68–74]. Essentially, when information sharing is active, upward members of the supply chain have two sources of information available: orders from right downward echelons and the end customer data. The way to deal with both sources of data is not unique. Some articles integrate both, modifying the previous forecasting models [42, 59, 72] while others simply replace orders by the end customer data without changing the forecasting technique [68–70]. The implications of integrating versus replacing still remains as an open question. Note that partial information sharing has also been investigated [69, 74, 75]. Viswanathan et al. [76] analyze the performance of a synchronized ordering scheme that only shares the ordering interval between echelons in a MRP framework. These authors remark the importance of coordination even when the lack of trust may complicate the sharing of sensitive information.

Apart from sharing end customer demand data, Cannella et al. [43] analyze different cases where inventory information was shared, Chen and Lee [77] identify the projected future orders of the downstream member as a key variable for information sharing. Although most of the information shared went from downward to upward members, Lee and Whang [63] explain how sharing order status from upward to downward members can help solve first call problems.

### 4.2 Type of research

The analysis of how information sharing has reduced the BWE has been carried out under different types of research. The most common approach (51 out of 73, 70%) is the theoretical one. In this type of research, simulations are usually employed, although under many different software systems. Originally, a continuous simulation system called DYNAMO was employed [13, 14], Chatfield et al. [69] created a *k*-stage serial supply chain simulation model (SISCO “Simulator for Integrated Supply Chain Operations”) that was employed to analyze the impact of information sharing. Fiala [78] suggested STELLA software. SIMULINK/MATLAB was employed by [51, 79]; AweSim 3.0 by [42]; Rockwell Software Arena v5.0 by [75]; Vensim PLE by [43]; SIMUL8 by [74]; *iThink*^®^ by [80]. An extension of the Beer Distribution Game applied to reverse supply chains is utilized as the Cider Game in [81]. In the rest of theoretical studies, either a general scientific software was employed or it was not clearly specified.

Apart from the theoretical simulations, where Bullwhip causes are analyzed from an operational perspective, the Bullwhip causes can also be studied from a behavioral point of view. The behavioral type of research is the second most investigated with a total of 17 articles (23%). In this case, usually, the forecasting and ordering policies are judgmentally introduced by human agents, although they can also be replaced by automatic decisions [82]. Bullwhip causes are also investigated by means of simulation games. The most well-known simulation game is the Beer Distribution Game [56, 57, 83], although other simulation game versions are employed. For instance, the Québec wood supply game, [61, 84, 85] or the Lean Leap Logistics Game, [86], derived from the Lean Processing Program related to a problem of product conversion in the automotive steel supply chain in the UK.

Empirical works are scarcer than theoretical ones. Trapero et al. [59] analyze the influence of information sharing on the forecasting accuracy using real sales and shipments data between two serially linked companies located in the UK within the personal care industry. Yao and Zhu [87] study empirically the effect of information sharing on the BWE using macroeconomic industrial data in the US. Wong et al. [42] compare the BWE decrease whether retailers share or not certain downstream information in a three-level (divergent) toy supply chain subject to seasonal demand. In this article, forecast updating mechanism and replenishment rules are modified to accommodate shared information. Croson and Donohue [83] examine the influence of point of sales data on the ordering decision and how it can reduce the BWE. McCullen and Towill [88] explain how to reduce BWE using rapid response manufacturing, putting in place a Distribution Requirements Planning (DRP) in precision mechanical engineering sector and supply chain integration. Furthermore, they reduced the material processing lead time from 23 to 2 weeks (time compression of 91%) and the BWE up to 58%.

Finally, 6 out of 73 articles utilize a descriptive approach. In this class of articles, authors describe the reality of the collaboration between companies based on how they are currently working. Among the descriptive articles, references [22, 23, 25, 34] are either surveys or state of the art works that have been mentioned in the introduction. Lee and Whang [63] described the types of information shared in several industry examples, as well as the alternative technological systems to enable information sharing. A case study in the supply chain of The Absolut Company is investigated in [89]. In that reference, the authors demonstrate the relationship between reciprocal and serial interdependence and IE patterns in dyads. They also analyze the barriers of sharing information across multiple tiers given pooled interdependencies.

### 4.3 Supply chain structure and number of echelons

The supply chain structure in IE is mainly serial (76%), followed by a divergent structure. On this subject, the number of echelons analyzed in those supply chain structures tend to be uniform, where frequently, the articles analyzed supply chains with 4 or more echelons.

### 4.4 Type of demand and forecasting model

The adequate forecasting model/method depends on the demand that the company is facing. Such a demand can be classified according to how it is generated, i.e., either simulated (artificially generated with random numbers following a statistical distribution) or real (obtained from a company). Most of the articles considered a simulated demand (82% approx.). Among the demand types, we can find stationary demands that follow the independent, identically and distributed (iid) assumption, for example [44, 69, 74, 85]. In these cases, the typical forecasting technique employed was moving average and single exponential smoothing. Several works employ stationary but correlated demands as AR(1) [70, 71, 77]. Non-stationary demands are also analyzed [71–73, 77, 90]. Reference [77] employed a generalized demand model, particularly, the Martingale model of forecast evolution that incorporates particular demand models as the iid normal demand model, the AR(1) model, the IMA(0,1,1) model, the general ARMA model, the linear state-space model and the advance demand information model. In addition, works centered on inventory policies employ a different type of demand typically used in a control engineering setting as step inputs [44] and pulse inputs [43] to excite the system. It is worth noting that more evidence is necessary to see the potential of machine learning forecasting techniques, since only 2 articles referred to them in this literature review, [59, 91]. Furthermore, fast moving consumer goods are the most common items analyzed, while slow moving demand items are very scarce, where only reference [76] analyzed these products by means of Croston method.

Despite the fact that demand forecasting in companies still relies on judgmental adjustments [92, 93], a rigorous analysis of the relationship between judgmental forecasting and IE has not been identified. In other words, would suppliers’ demand planners benefit from accessing end customer sales data to judgmentally adjust demand forecasts? Such a kind of question remains open and it suggests an interesting stream of research.

### 4.5 Inventory control technique and assumptions

OUT policies are the most frequently used (47% approx). The second most frequent inventory policy is based on a control engineering perspective. For example, the family of smoothing replenishment rules [79] based on a control engineering perspective. Authors in [94] and [77] have proposed generalized versions of OUT policy, where Hosoda and Disney [94] analyze the effect of a proportional controller incorporated into the OUT policy and Chen and Lee [77] is based on the Martingale model of forecast evolution for the demand process. Ouyang and collaborators in [71, 95] analyzed a broad family of ordering policies that are both proper and linear and time-invariant, for instance, OUT policies fulfill those conditions.

Order policies such as EOQ and JIT are also present with a few examples. Viswanathan et al. [76] use a dynamic lot-sizing technique based on the Silver-Meal heuristic [58] to calculate the economic order interval; downstream echelons only were allowed to place orders at fixed interval (or *n* x economic order interval). Lee and Kumara [96] evaluate and compare different types of replenishment policies among themselves (i) Standard Branch-and-Bound method; (ii) Wagner-Whitin algorithm proposed by [97]; and three initial plans used as benchmarks: (iii) Latest Order Planning; (iv) Earliest Order Planning; and (v) Upstream Planning always satisfying the external demand. The aim was to minimize the cost plan.

Nienhaus et al. [82] allow users to simulate the beer game playing with the computer. In this regard, it is possible to define techniques such as (i) Moving average/standard deviation where the amount ordered is the average orders during the last five periods plus an amount to cover the safety stock based on the standard deviation, (ii) Keep level of stock, which was the best solution, where orders received from a customer are passed on to the supplier; and human-like strategies: (iii) Safe harbour, where more than what is actually necessary is ordered and then safety stock is increased; and (iv) Panic, where stock is emptied before the end customer’s demand increases. Hieber and Hartel [98] use 13 techniques split in different groups: Standard strategies (Re-order quantity and EOQ), Random order strategies (basic, panic and limited), Matching demand and supply strategies (basic, panic and limited), history order strategies (basic, panic and limited) and interactive strategy.

Another stream of ordering rules is the one proposed in [61, 84, 85], which is based on tokens to achieve a decentralized coordination validated by the Wood Supply Game, where companies in the supply chain are considered agents. The idea behind that is to divide the orders in two parts: i) the classical order to manage real demand; ii) a token to manage fluctuations of such orders.

It is interesting to note that most of the inventory control techniques that assume stochastic demand also assume total backordering, where lost sales assumption is only made in 4 articles.

Although the implementation of an IE framework aims to improve the forecasting performance, some articles analyze the impact of information sharing on the replenishment policy used. In this sense, as occurred with the forecasting models, some works modify the replenishment policy to incorporate the shared information efficiently, [42–44].

### 4.6 Collaboration objectives and Bullwhip effect

The most frequent objective of collaboration is to analyze the BWE, with almost 62% of the articles. The BWE is reduced in most of them. Nonetheless, some articles [99, 100], state an increase of the BWE. Authors in [99] investigate the typical assumption of permitting returns (negative orders) and they show that significantly increases the BWE. Thonemann [100] analyzes the fact of sharing Advance Demand Information (ADI). Customers share both aggregated and detailed ADI with manufacturers. Although such information exchange can reduce the cost of the manufacturer, it may also increase the variability of the production, thus increasing the BWE.

Apart from reducing the BWE, the rest of objectives are also documented in the IE category, where customer service level is the less frequent among the collaboration objectives.

## 5 Type II—Vendor managed replenishment

**Fig 6:**
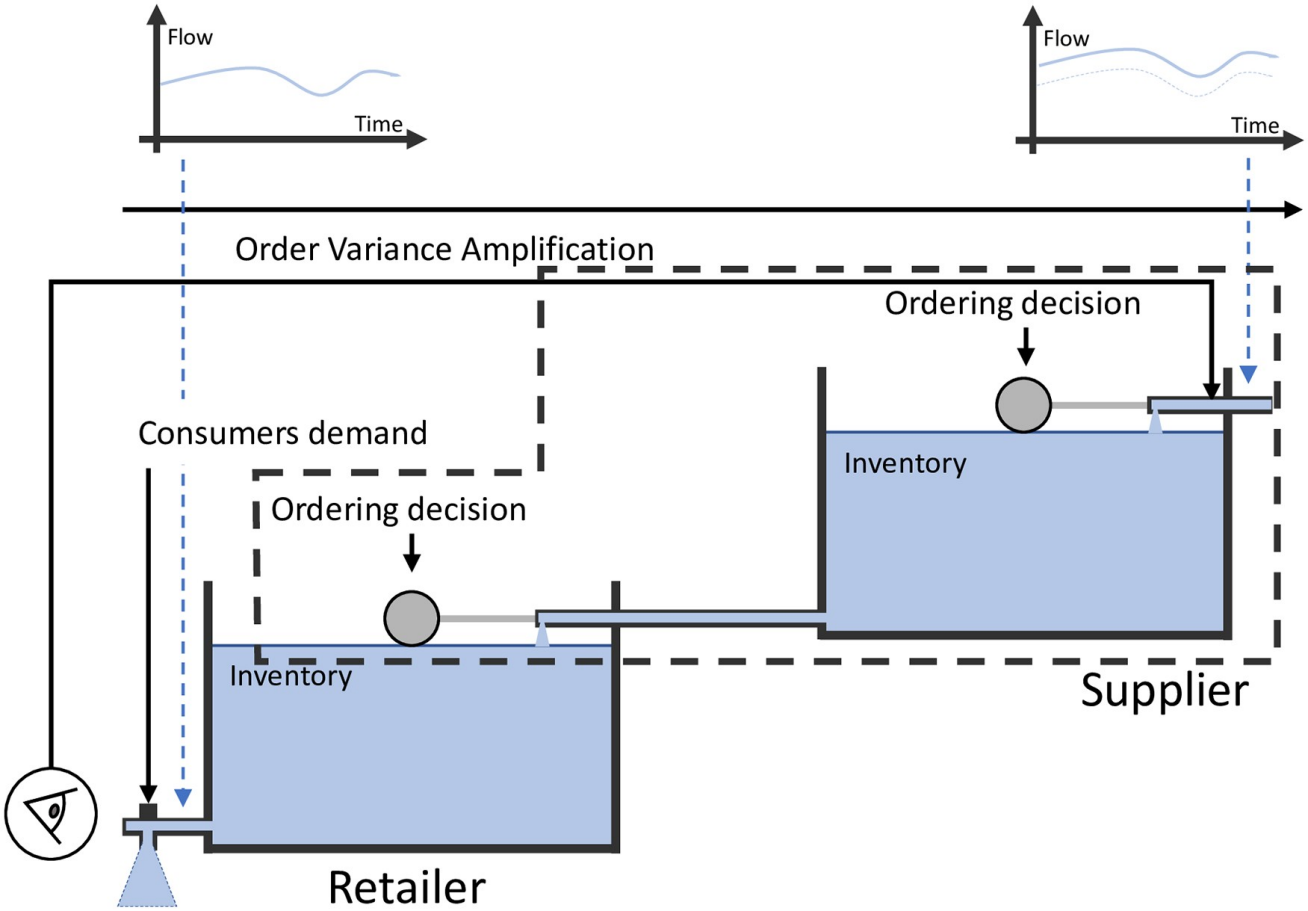
Type II—Vendor managed replenishment.

According to [34], “*Vendor Managed Replenishment means that the task of generating the replenishment order is given to the supplier, who takes the responsibility for maintaining the retailer’s inventory, and subsequently, the retailer’s service levels […] having full visibility of the stock at the customer’s site, the supplier is wholly responsible for managing the inventory*.” Fig 6 exemplifies the VMR using the water-tank analogy, where the retailer ordering decision is within a box in a dashed line together with the supplier ordering decision. That represents that the supplier is in charge of the retailer ordering decision. Note that the upper plots of flow vs. time are not identical, because they are not synchronised, but they show the same demand variability. That indicates that VMR can remove completely the BWE. The vendor can be the supplier in a retailer-supplier collaboration, although the analysis is applicable to the wholesaler-distributor or the distributor-manufacturer relationships as well. Vendor and customer create a mutually agreed framework of trust, where vendor monitors and constantly updates the performance targets creating an environment of continuous improvement [101, 102], eliminating one layer of decision-making and information flow time delays [51].

VMR is the second step in the collaboration stairs, see Fig 1, since it cannot take place without information sharing. It is also the second most analyzed with 18 out of 92 articles in our sample, see S3 Table.

One of the problems the authors found was the acronyms puzzle of different initiatives related to VMR. Table 2 shows the different acronyms and the references where they were found. Regarding that table, note that authors do not consider Consignement Stock (CS) a VMR collaboration approach, in line with [34]. Instead, in our view, it is closer to a traditional supply chain, where the differentiating attribute is that stock is stored in the customer’s facilities, although the owner is the supplier and customers only pay when the product is consumed, and thus, decisions are made independently. Furthermore, CPFR is considered as a Synchronized Supply Chain rather than a VMR.

**Table 2:** List of acronyms that have been used to mention VMR collaboration types.

Name	Acronym	References
Advanced Continuous Replenishment	ACR	[103]
Collaborative Commerce	CC	[104]
Centralized Inventory Management	CIM	[51, 52, 105]
Co-managed Inventory	CMI	[104]
Continuous Partnerships	CP	[103]
Collaborative Planning, Forecasting and Replenishment	CPFR	[51, 52, 104–106]
Continuous Replenishment	CR	[32, 51, 52]
Continuous Replenishment Program	CRP	[11, 105, 107]
Consignment Stock	CS	[25, 108]
Collaborative Transport Management	CTM	[104]
Efficient Consumer Response	ECR	[32, 51, 52, 104–106]
Efficient Food-service Response	EFR	[104]
Quick Response	QR	[51, 52, 103]
Quick Response Manufacturing	QRM	[34]
Rapid Replenishment	RR	[51, 52, 105]
Synchronized Consumer Response	SCR	[51, 52, 105]
Vendor Managed Inventory	VMI	[11, 25, 32, 34, 51, 52, 103–113]
Vendor Managed Replenishment	VMR	[34]

### 5.1 Type of information exchanged

The information usually shared to set the VMR are demand, measured by either orders or sales, and inventory. 72% of the articles have both inputs [25, 32, 51, 52, 103, 105, 106, 108–113]. Holweg et al. [34] consider only the stock information at the customer’s site as information required and Xu et al. [107] consider sufficient the retailer’s actual demand information as the base of collaboration.

Apart from the information shared, how such information is sent to the supplier is also an important matter. In fact, Xu et al. [107] argue that this interface should not be disregarded, since it is one of the critical parts of the forecasting process. New information technologies as internet or RFID have been used to foster such sharing [104]. Unfortunately, just a few articles provide this kind of details. In general, it can be sent via (i) EDI [107, 112]; and (ii) ICTs [32, 103, 104, 106, 110, 113]. Additionally, SAP interface is mentioned in [25].

### 5.2 Type of research

Most of the articles are theoretical (61%). 4 out of these 11 use MATLAB to carry out the simulations [51, 52, 105, 108], and reference [109] uses GoldSim simulation software.

6 articles out of 18 are descriptive [11, 25, 32, 34, 52, 104]. 2 out of these 6 articles [52, 104] expose mainly the BWE problem and causes. Olson and Xie [32] compare different coordinated supply chain inventory management systems and review several simulation VMI studies. Kouvelis et al. [11] is a survey that indicates the contribution of Productions and Operations Management journal on SCM. One of the topics included is supply chain coordination, where schemes such as VMR are discussed.

2 articles out of 18 are empirical [110, 112]. Kaipia et al. [110] evaluate the performance of a manufacturer planning process within the electronics industry. They give some advice to improve As-Is VMI model when a new product is released. Dong et al. [112] examine benefits to downward members from implementing a VMI with respect to an IE collaboration type. This work uses an item-level data set comprised of 30 distributors and one manufacturer. 20 out of 30 distributors have implemented a VMI in different time periods, whereas 10 had just implemented an IE. Both implementations were based on a third-party information-services provider. Two econometric models were assessed to compare inventory, stock-out levels and inventory variability for items managed with VMI with respect to items managed with IE. The results showed that VMI reduced inventory levels by 7% on average, stock-outs by 31%, and inventory variability by 9%.

Finally, 1 article is behavioral [106] and concludes that BWE is not reduced using a VMI strategy. The conclusion is based on the fact that, although ICTs provide transparency and reduce drastically the uncertainty, they create a much more complex environment with a lot of additional information that the decision-maker can hardly manage.

### 5.3 Supply chain structure and number of echelons

The most common supply chain structure used in VMR articles is a linear two-echelon supply chain, 7 articles out of 18. Another stream, 3 out of 18, use a divergent two-echelon supply chain [108, 112, 113]. Kaipia et al. [110] use a divergent four-echelon supply chain, a two-echelon network supply chain is used in [103], and a linear four-echelon supply chain in [75, 106].

### 5.4 Type of demand and forecasting model

Unlike information sharing, decisions about forecasting technique and replenishment policy are less relevant with regards to the subject of the articles. Only 27% of the articles provide information about the forecast technique applied and they all utilized exponential smoothing, the rest of the articles do not provide clear information about the technique used. Note that most of the demand patterns are simulated, where only 3 articles [34, 110, 112] deal with real data.

### 5.5 Inventory control technique and assumptions

Regarding replenishment policies, 5 works use Automatic Pipeline, Inventory and Order Based Production Control System (APIOBPCS) [51, 52, 105, 106, 111]. Other 4 use OUT [52, 75, 107, 109]. Disney and Towill [52] compare APIOBPCS against OUT in order to evaluate its performance. A complex heuristic algorithm called particle swarm optimization to solve the problem is used by [103]. The rest of the articles, 9, do not provide any relevant specific information.

Due to the long lead time, Chen et al. [113] use a transshipment point to move material among the retailers, based on their sales in the previous period.

The most common inventory assumption was total backordering, where only 3 articles [103, 108, 113] assumed lost sales case. Note that Chen et al. [113] use backlogging during the first period and lost sales only in the second part of the experiment.

### 5.6 Collaboration objectives and Bullwhip effect

Regarding the objectives pursued in the VMR articles, Sadeghi et al.[103] minimized the total inventory cost along the supply chain. Supplier production cost and inventory and retailer purchase costs are analyzed in [51, 105, 112]. Shortage, holding and order costs are evaluated in [75]. Considering inventory level, Xu et al. [107] focus on reducing safety stocks as an important part on the level of inventories held by a company. Disney et al. [106] aim to reduce the total system inventory level. Kristianto et al. [109] reduce inventory standard levels and the magnification of the order variance. Sadeghi et al. [108] aim to find the optimum order size, the replenishment frequency of the retailers and the shortest route, minimizing total inventory and transportation cost. Kaipia et al. [110] reduce the maximum inventory level target in the retailer when a new product is released.

Focusing on consumer service objective, supplier distribution service level and retailer product fill rate are studied in [34]. Maintaining customer service level reducing inventory investment is also investigated in [111]. Yu et al. [75] also investigate fulfillment rate and customer service level.

Regarding the BWE, 12 out of 18 analyze it either as the main objective [52] or as a secondary one, and confirm that using a VMR strategy reduces the BWE.

## 6 Type III—Synchronized supply chain

**Fig 7:**
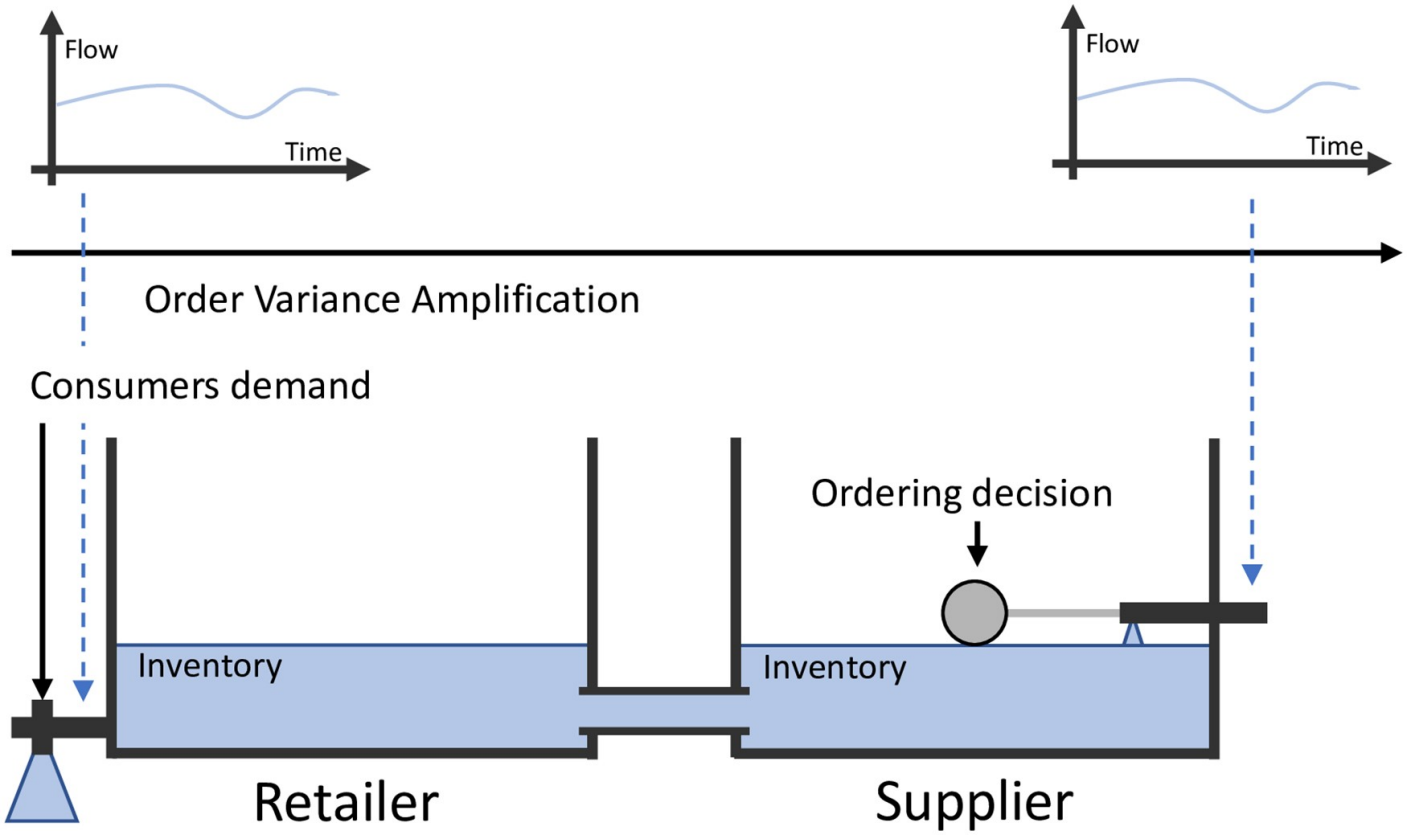
Type III—Synchronized supply chain.

According to reference [34], this type of collaboration eliminates one decision point and merges the replenishment decision with the supplier’s production and materials planning. Supplier not only takes charge of the customer’s inventory replenishment at operations level, but also uses the visibility when planning its own supply operations. The benefit is that downstream requirements are smoother than purchase orders generated based on a reorder role and, the demand variability, stock cover and the costs become lower [114]. Sari [39] argues that SSC, also called CPFR, requires all members of a supply chain to jointly develop demand forecasts, production and purchasing plans, and inventory replenishment. Following the water-tank analogy, Fig 7 represents the SSC, where the main difference with respect to the previous collaboration schemes is that the planning and inventory processes are centralized and that is represented by linking the two tanks together. That means that the two tanks are driven by the retailers demand and they are synchronized by a single ordering decision. This is also represented by the identical upper plots of flow vs. time. S4 Table shows the summary of the SSC typology.

### 6.1 Type of information exchanged

Unlike VMR, SSC utilizes more information to build the model: (i) Member’s orders [43, 60, 88]; (ii) inventory levels [43, 60, 88, 115–117]; (iii) work in progress levels [43, 60, 116]; (iv) lead times [43, 60, 118]; (v) safety stock factors upstream [60]; (vi) market demand information [43, 60, 88, 115, 117]; (vii) demand forecast [43, 88, 117]; (viii) Master Production Schedule (MPS) [88]; (ix) replenishment orders [43, 88, 117]; (x) promotion information [115, 117]; (xi) capacity information [115]; (xii) sales plan [116]; (xiii) lead time of work orders [116]; (xiv) demand patterns [118]; (xv) replenishment policies [118]; (xvi) parameter settings [118]; (xvii) delivery information [43, 117]; (xviii) supplier item shortage [117]; (xix) competitor information about promotion information or marketing strategy [117]; and (xx) backlog information [43].

### 6.2 Type of research

Classifying the articles by type of research, 6 out of 11 articles are theoretical [43, 60, 115, 117–119]; 3 articles are classified as empirical [88, 116, 117], where [117] can be classified as both theoretical and empirical works; and finally, references [32, 34] are categorized as descriptive. Note that any behavioural work has been found when dealing with SSC.

### 6.3 Supply chain structure and number of echelons

The most typical is a linear two-echelon supply chain, with 4 out of 11 articles [34, 115, 117, 118]. The following one is a linear four-echelon supply chain with 3 [43, 60, 120]. McCullen and Towill [88] use a divergent three-echelon supply chain and a network of four-echelons is represented in [116].

### 6.4 Type of demand and forecasting model

The forecasting techniques employed are entirely univariate based on either moving average [117] or exponential smoothing [43, 117, 120]. The demand types employed are well-balanced with 5 articles working with simulated demand and 4 with real demand.

### 6.5 Inventory control technique and assumptions

Only 6 articles provide information about the replenishment technique used: (i) smoothing replenishment policies [60]; (ii) Synchronized Base Stock policies (SBS) [116]; (iii) OUT level [115, 118]; (iv) fixed time period policy [117]; and (v) Deziel and Eilon Automatic Pipeline Variable Inventory and Order-Based Production Control System (DE-APVIOBPCS) [43]. All of them assume total backordering and none includes the lost sales situation.

### 6.6 Collaboration objectives and Bullwhip effect

The collaboration objectives found are: (1) reducing inventory level by either reducing the safety stock (a safety buffer is always required)[34, 88] or the stock and obsolescence [116], (2) increasing the customer service levels improving the fill rate [32, 60, 88, 115, 116], (3) reducing overall cost of the supply chain [32, 43, 117, 118].

Focusing on the BWE, it was the main primary objective in [43] and [60, 88, 115–119] as a secondary. All of them confirmed that SSC helps eliminate BWE.

## 7 Conclusion

Supply Chain Collaboration as a tool to reduce the Bullwhip effect and associated costs is an attractive work area for practitioners and academics alike. However, novel developments under different acronyms are spread in the literature making very difficult to track potential advancements. This work intends to organize such developments by classifying the published articles according to 3 types of collaboration mechanisms: Information Exchange, Vendor Managed Replenishment and Synchronized Supply Chain. The bibliography was selected following a systematic literature review process, where 92 articles were finally considered. Those articles were analyzed to extract their main operational characteristics as type of research, collaboration objective, supply chain structure, type of demand, forecasting model, inventory control technique and respective assumptions. Those features were summarized in 3 tables.

In general, Information Exchange is the collaborative scheme better studied with 73 articles, followed at some distance by Vendor Managed Replenishment (18) and finally Synchronized Supply Chain (11). The main conclusions/gaps referring to operational decisions that can be drawn across the different types of collaboration are: i) given the complexity of modeling supply chains, the simulation approach is the preferred one. Nonetheless, given the plethora of simulation software available for both theoretical and behavioral experiments, developing a free supply chain toolbox in simulation software would be of great benefit. This toolbox would allow researchers to either reproduce or use previous models, which have been tested, so that they do not have to implement it from scratch and avoid mistakes in the implementation; ii) there exists a serious lack of empirical works at various levels. For example, fully empirical works as [59, 87] and semi-empirical works where real demand data is employed within simulations to verify theoretical developments as [68, 121]; iii) the integration versus substitution issue of end demand as well as other demand drivers in relation to the forecasting model still remains an open question and further research is needed about the adequacy of each option; iv) the influence of shared information on the accuracy of judgmental forecasting is completely overlooked; v) more comparisons between machine learning and traditional statistical forecasting methods is also required; and finally, vi) despite the fact that lost sales case is frequent in some sectors as retailing [122], most of the simulations are based on the total backordering assumption and more research is needed assuming the lost sales case.

Although the previous conclusions apply to the 3 types of collaboration, as we move forward to more sophisticated extents of collaboration, some of the aforementioned weaknesses become more exacerbated. For instance, since suppliers have access to enriched information (end customer sales data, retailer inventory level, etc.), they must consider using more complex forecast techniques. As authors have disclosed, most of the theoretical articles use exponential smoothing as forecasting technique. The same conclusion can be extended to inventory policies. The inclusion of machine learning approaches in complex collaborative mechanisms is a potential research opportunity. Additionally, most of the articles use serial dyads, this approach is really distant from practitioners #x2019; reality, widening the gap between real practice and theory.

In relation to Synchronized Supply Chain, it should be noted the scarce number of works. In fact, no behavioral research was found and inventory assumptions are only based in total backordering assumption without exploring the lost sales case. Generally speaking, companies that tried, are trying or will try to implement such a collaboration need to understand the complexity and potential problems that they need to cope with: (i) difficulty to properly incorporate customer information into the supplier systems and not only as an input into the judgmental decision process [32, 34, 116–118], (ii) distance between supplier and customer becomes important because the lead time [34], (iii) lack of “real-time” information in the processes causing higher level of stock [88, 115].

Apart from the aforementioned operational difficulties associated to collaborative mechanisms, it is also important to consider behavioural issues such as the lack of trust between supply chain partners and the risk of information leakage [123].

It is also important to get a deeper insight about implementation issues, since it is a cumbersome task for suppliers to handle clients with and without collaborative mechanisms at the same time. Despite the implementation difficulties, achieving a collaborative program as vendor managed replenishment seems to be a win-win strategy to pursue. In the short/mid-term, supplier looks like the main beneficiary since the benefits in safety stock and resource waste reduction are more tangible, but retailer does not incur in costs related to demand forecasting, order placement and excessive inventory held, providing strong incentives to join supplier in such initiative. Long-term, supplier can retain customers building loyalty and reducing costs using the information shared and the retailer can ensure an interrupted supply of the product to keep operations going, whilst inventory level is reduced and, therefore, labor costs. Nevertheless, the way benefits should be shared among the members of the collaboration is also a promising further research.

It is the first author’s view that, as a practitioner in several projects related to implement collaborative mechanisms between companies, nowadays, for many organizations, Synchronized Supply Chain implementation is still assessed as a pilot project that needs to be further developed in order to convince managers to invest on it. Hopefully, the present literature review will guide researchers and practitioners to develop further insights in this exciting discipline.

## Supporting information

S1 Table List of the 92 articles included in the analysis [124–150]. This list is organised by importance according to the number of citations. This organization of articles is utilized for the following tables.
(PDF)

S2 Table IE summary. (PDF)

S3 Table VMR summary. (PDF)

S4 Table SSC summary. (PDF)

S1 Appendix Search commands employed. (PDF)

S1 Checklist (PDF)

S1 Fig (PDF)
