# Supplementary material for: A literature review on operational decisions applied to collaborative supply chains
Source: PLoS One. 2020 Mar 13;15(3):e0230152. doi: 10.1371/journal.pone.0230152 (PMC7069626; doi:10.1371/journal.pone.0230152)
Supplement: S1 Table — This list is organised by importance according to the number of citations. This organization of articles is utilized for the following tables. (PDF) [file pone.0230152.s001.pdf]

S1 Table. List of the 92 articles included in the analysis. This list is organised by importance according to the number of citations. This organization of articles is utilized for the following tables.

No	Reference	No	Reference	No	Reference
1	[16]	32	[68]	63	[112]
2	[34]	33	[56]	64	[72]
3	[51]	34	[22]	65	[73]
4	[124]	35	[125]	66	[113]
5	[126]	36	[57]	67	[127]
6	[78]	37	[88]	68	[90]
7	[69]	38	[25]	69	[61]
8	[63]	39	[59]	70	[23]
9	[11]	40	[115]	71	[84]
10	[104]	41	[121]	72	[98]
11	[52]	42	[119]	73	[44]
12	[83]	43	[103]	74	[117]
13	[107]	44	[128]	75	[129]
14	[106]	45	[109]	76	[130]
15	[91]	46	[131]	77	[132]
16	[70]	47	[133]	78	[76]
17	[100]	48	[99]	79	[134]
18	[86]	49	[108]	80	[135]
19	[105]	50	[94]	81	[136]
20	[137]	51	[81]	82	[80]
21	[138]	52	[116]	83	[139]
22	[140]	53	[82]	84	[87]
23	[77]	54	[118]	85	[141]
24	[142]	55	[110]	86	[143]
25	[71]	56	[144]	87	[32]
26	[94]	57	[43]	88	[145]
27	[146]	58	[74]	89	[147]
28	[60]	59	[148]	90	[89]
29	[75]	60	[149]	91	[96]
30	[85]	61	[150]	92	[42]
31	[95]	62	[111]		
