# Supplementary material for: A literature review on operational decisions applied to collaborative supply chains
Source: PLoS One. 2020 Mar 13;15(3):e0230152. doi: 10.1371/journal.pone.0230152 (PMC7069626; doi:10.1371/journal.pone.0230152)
Supplement: S3 Table — (PDF) [file pone.0230152.s003.pdf]

S3 Table. VMR summary.

Dimension	Intercompany integration			Type of research				Number of echelons				Supply chain structure					Type of demand			Forecasting technique							Replenishment policy							Inventory assumptions			Collaboration objective						Bullwhip effect			
Articles	IE	VMR	SSC	Empirical	Theoretical	Behavioural	Descriptive	2	3	4+	N/A	Serial	Network	Convergent	Divergent	N/A	Simulated	Real	N/A	ARIMA	Moving Average	Exp. Smoothing	Linear Regression	Machine learning	Other	N/A	Classical OUT	Control Engin.	Beer game	EOQ	JIT	Other	N/A	Backordering	Lost-sales	N/A	BWE	Uncertainty	Customer service	Inventory level	Reduce costs	N/A	Reduced	Non reduced	Statements of facts	N/A
2	x	x	x				x	x				x						x								x							x			x			x						x	
3		x			x			x				x					x									x		x								x	x				x		x			
9		x					x				x					x			x							x							x			x						x			x	
10		x					x				x					x			x							x							x			x						x			x	
11		x			x		x	x				x							x			x					x	x								x	x						x			
13		x			x			x				x					x					x					x							x			x			x			x			
14	x	x			x	x				x		x					x					x						x						x			x			x			x			
19		x			x			x				x					x					x						x						x			x				x		x			
29	x	x			x					x		x					x									x	x									x	x		x		x		x		x	
38	x	x	x				x				x					x			x							x							x			x						x				x
43		x			x			x					x				x									x						x			x						x					x
45		x			x			x				x					x					x					x							x			x			x			x			
49		x			x			x							x		x									x							x		x		x			x			x			
55		x		x						x					x			x								x							x			x	x			x			x			

Articles	IE	VMR	SSC	Empirical	Theoretical	Behavioural	Descriptive	2	3	4+	N/A	Serial	Network	Convergent	Divergent	N/A	Simulated	Real	N/A	ARIMA	Moving Average	Exp. Smoothing	Linear Regression	Machine learning	Other	N/A	Classical OUT	Control Engin.	Beer game	EOQ	JIT	Other	N/A	Backordering	Lost-sales	N/A	BWE	Uncertainty	Customer service	Inventory level	Reduce costs	N/A	Reduced	Non reduced	Statements of facts	N/A
62		x			x			x				x					x									x		x						x			x		x				x			
63	x	x		x				x							x			x								x							x	x			x				x		x			
66		x			x			x							x		x									x							x	x	x		x		x		s		x			
87		x	x				x				x					x			x							x							x			x						x			x	
