# Supplementary material for: A literature review on operational decisions applied to collaborative supply chains
Source: PLoS One. 2020 Mar 13;15(3):e0230152. doi: 10.1371/journal.pone.0230152 (PMC7069626; doi:10.1371/journal.pone.0230152)
Supplement: S1 Appendix — (PDF) [file pone.0230152.s005.pdf]

S1 Appendix. Search commands employed. The filter applied to select the articles has two differentiated parts. The first one focused on the collaboration models:

((TITLE-ABS-KEY (supply AND chain AND collaboration) OR TITLE-ABS-KEY (collaborative AND model) OR TITLE-ABS-KEY (collaborative AND supply AND chain) OR TITLE-ABS-KEY (supply AND chain AND coordination) OR TITLE-ABS-KEY (information AND sharing) OR TITLE-ABS-KEY (supply AND chain AND integration) OR TITLE-ABS-KEY (vmi) OR TITLE-ABS-KEY (vmr) OR TITLE-ABS-KEY (vmi) OR TITLE-ABS-KEY (cpfr) OR TITLE-ABS-KEY (ecr) OR TITLE-ABS-KEY (information AND exchange)))

and the second one referring to BWE:

AND ((TITLE-ABS-KEY (bullwhip AND effect) OR TITLE-ABS-KEY (forrester AND effect) OR TITLE-ABS-KEY (bullwhip) OR TITLE-ABS-KEY (bullwhip-effect)))
